# Inflammation, Glutamate, and Cognition in Bipolar Disorder Type II: A Proof of Concept Study

**DOI:** 10.3389/fpsyt.2019.00066

**Published:** 2019-03-01

**Authors:** Sinead King, Luke A. Jelen, Charlotte M. Horne, Anthony Cleare, Carmine M. Pariante, Allan H. Young, James M. Stone

**Affiliations:** ^1^Centre for Affective Disorders, Department of Psychological Medicine, Institute of Psychiatry, Psychology and Neuroscience, King's College London, London, United Kingdom; ^2^South London and Maudsley NHS Foundation Trust, London, United Kingdom; ^3^Department of Psychosis Studies, King's College London, London, United Kingdom; ^4^Centre for Neuroimaging Sciences, Institute of Psychiatry, Psychology and Neuroscience, King's College London, London, United Kingdom

**Keywords:** TNFa, cognition, glutamate, bipolar disorder type II, inflammation

## Abstract

**Background:** Two current theories regarding the neuroscientific bases of mood disorders involve alterations in glutamatergic neurotransmission and excessive activation of inflammatory pathways. We hypothesized that glutamate (Glu) levels and peripheral inflammatory markers would be associated with cognitive function, in patients with Bipolar Disorder Type II (BP-II), and that such factors would be associated with psychological treatment outcomes.

**Aims:** The primary aim of this study was to explore the relationship between the neurotransmitter Glu, cytokines (CRP, IL_6, and TNFa) and neuropsychological and related functioning. The secondary aim was to assess cognitive functioning as a predictor of poor response to psychological therapy.

**Methods:** Proton magnetic resonance spectroscopy data were acquired from the anterior cingulate cortex (ACC) of 15 participants with BP-II, and 13 healthy controls in a 3T magnetic resonance imaging scanner. The Digit Symbol Task (DST) for processing speed, TMT-B for executive function and Rey Auditory Verbal Learning Test (RAVLT) were administered to assess cognitive domains.

**Results:** There was no significant difference in anterior cingulate Glu, or inflammatory markers between groups. Furthermore, we found no significant difference between groups in any cognitive tests. Scores on the DST were found to be significantly associated with poor response to psychological therapy.

**Conclusions:** This study may highlight an association between neuropsychological dysfunction and treatment outcome in euthymic patients with BP-II. We did not find any association between peripheral inflammatory markers and brain Glu levels. This may have been in part due to the small sample size.

## Introduction

Mood disorders are highly prevalent and represent a significant public health burden (1). Psychological treatments are thought to be effective modes of treatment for depression and anxiety, yet there are a substantial number of patients who do not benefit (2). It is therefore possible that underlying biological or neuro-psychological mechanisms are involved in preventing patients from fully benefiting from therapy. Due to the severity and co-morbidity of treatment resistant populations, it is likely that patients are not receiving the right treatment to suit their individual needs. To address this problem, more refined approaches are needed to target specific biological and cognitive domains. Two current areas of focus regarding the neuroscientific basis of mood disorders involve alterations in glutamatergic neurotransmission and excessive activation of inflammatory pathways (3).

Since glutamate mediates cognition and behavior (4) and the predominant role of glutamate receptors appears to be the modulation of synaptic plasticity, a property of the brain thought to be vital for memory and learning (5), it is plausible that dysregulation of the glutamatergic system may be involved in treatment resistant mood disorders. Disruption of this system has been consistently implicated in psychiatric illnesses, including major depressive disorder (MDD) and bipolar disorder (BP), in multiple brain areas using proton magnetic resonance spectroscopy (^1^H-MRS) (3, 6, 7). This technique allows *in vivo* assessment of the chemical composition of tissues in a non-invasive manner, by using the magnetic resonance signal of hydrogen to determine the concentrations of various metabolites, including glutamate.

Studies using single voxel ^1^H-MRS in the anterior cingulate cortex (ACC), in MDD have consistently reported lower glutamatergic metabolites glutamate (Glu), glutamine (Gln), or Glx (Glu+Gln) (8–12). Studies in bipolar disorder have demonstrated inconsistent results but suggest that levels may be elevated (9, 10, 13). Such inconsistencies may be due to the difference in mood phase between various studies. For example, patients with mania show elevated Glx levels in the left dorsolateral prefrontal cortex (DLPFC) compared with healthy subjects (14). Considering the role of glutamate between subtypes of bipolar disorder (i.e., type I vs. II), Atagun et al. found neurochemical differences in superior temporal cortices (15), i.e., BP-I had significantly lower Glu and glutamate+glutamine (Glx) levels in comparison to BP-II. However, this finding may be due to bipolar disorder type I consistently shown to be taking more anti-psychotic medication (16). Therefore, glutamate concentrations may provide information about neuronal function during a particular mood episode, but also reveal underpinnings that are common/distinct across types.

Indeed, the contribution to altered glutamate release/reuptake activation may be related to cytokine effects within the brain, leading to excitotoxicity and loss of glial elements, consistent with altered effects on cognition and behavior. Preliminary evidence shows that elevated inflammatory markers are associated with elevated glutamatergic neurotransmission (3), which subsequently correlated with poor processing speed. In work investigating how inflammatory markers may impact on the glutamatergic system, it has been shown that patients exposed to the inflammatory cytokine interferon (IFN)-alpha exhibited increased glutamate in left basal ganglia and dorsal anterior cingulate cortex (dACC) in a group of patients with hepatitis C and comorbid depression (17). In turn, high Glx, and Glx/creatine levels have been also been shown to correlate with cognitive impairment in various patient groups (18). A similar process may occur in bipolar disorder, since recent evidence shows elevated glutamate (10) and increased inflammatory markers have also been reported in this patient group (19), albeit in different patients. Increasing evidence is showing increased CRP, IL_6, and TNFa levels in euthymic bipolar patients in comparison to controls (20–22). Furthermore, elevated serum levels of IL-1RA in BP subjects, even during euthymic states, was found to be associated with worse cognitive function (23). Thus, there are some intriguing suggestions that there may be an interaction between inflammation, cognitive impairments and increased brain glutamate levels in BP that require further investigation (24).

The purpose of this study was three-fold. Firstly, we aimed to measure ACC glutamatergic concentrations at rest (i.e., static ^1^H-MRS) in bipolar disorder type II (BP-II) compared with healthy controls. Second, we aimed to examine any correlations with neuropsychological performance on cognitive tests, and peripheral inflammatory markers (CRP, IL_6, and TNF-a) to investigate cytokine involvement on the glutamatergic system and related effects on cognition and behavior. Lastly, we aimed to assess if the presence of neuropsychological impairment may in turn have subsequent effects on psychological treatment outcomes. We hypothesized that higher levels in Glu, would be associated with poorer cognitive performance in the BP-II group compared with healthy controls and this would be related to the inflammatory cytokines TNFa, CRP, and/or IL_6.

## Methods

### Study Subjects

Fifteen patients with BP-II and thirteen healthy controls aged 22–57 years were recruited into the study. The BP-II group were recruited from the Predictors of Outcome Following Psychological Therapy (PROMPT) study which aims to investigate predictors of response to psychological therapy (2). The PROMPT project is a large observational, naturalistic study which investigates predictors of outcome following a range of different psychological therapies, including cognitive behavioral therapy (CBT), guided self-help and counseling, at Southwark Talking Therapies, South London [for more information about the PROMPT study, see (2)]. Healthy controls were recruited through online advertisements. Bipolar patients were assessed for euthymia prior to testing, using short behavioral assessments to assess hypomania and depressive symptoms. However, three people were depressed at the time of their assessment. Exclusion criteria included any serious medical illness, or infection, and substance abuse/dependence within the past 6 weeks (determined by structured clinical interview for DSM-IV). No patients had taken any medications known to affect the immune system within the past 6 months. 5/15 participants were taking anti-depressant medications including citalopram and fluoxetine due to the difficulty in recruiting patients who were medication free. 10/15 patients were medication free. All participants signed informed consent and the study was approved a priori by the London—Harrow Research Ethics Committee, Ref 12/SC/0528.

### Study Procedures

Study procedures occurred in the same order over either 1 or 2 days. ^1^H-MRS scans were conducted on Day 2, if applicable, between 8 a.m. and 4 p.m. Blood sampling, behavioral assessments and neurocognitive testing were conducted on the same day as the brain scan, and neuropsychiatric assessments were carried out on Day 1 if the session was split into two. Psychological treatments for the BP-II group were delivered by a qualified psychologist or psychological well-being practitioner and all BP-II participants in this present study completed an average of six therapy sessions. Therapy outcome data was extracted from online patient records, as and when patients completed therapy. Depressive symptom severity, using the PHQ-9, is measured after each therapy session and at baseline. Outcome for the present study is defined as the final PHQ-9 score of the last therapy session received by each patient.

### Behavioral Assessments

Depression severity was measured using the Montgomery–Åsberg Depression Rating Scale (MADRS) (25). Hypomanic symptoms were assessed using the Young Mania Rating Scale (YMRS) (26). Therapy outcome scores for the 15 BP-II patients, in the form of the Patient Health Qusetionnaire-9 (PHQ-9), were obtained from the outcome phase of the PROMPT study in order to assess baseline predictors of therapy outcome in the present subsample of patients.

### Neurocognitive Assessment

Executive function was assessed using the Trail Making Test B and Intra Dimensional Extra Dimensional Shift of the Cambridge Neuropsychological Test Automated Battery. The Rey Auditory Verbal Learning Test (RAVLT) was used to measure verbal learning and memory, and the Digit Symbol Substitution Test of Wechsler Adult Intelligence Scale was used to assess psychomotor processing speed. The Weschler Test of Adult Reading was used to measure pre-morbid IQ.

### Scanning Protocol

**Figure 1:**
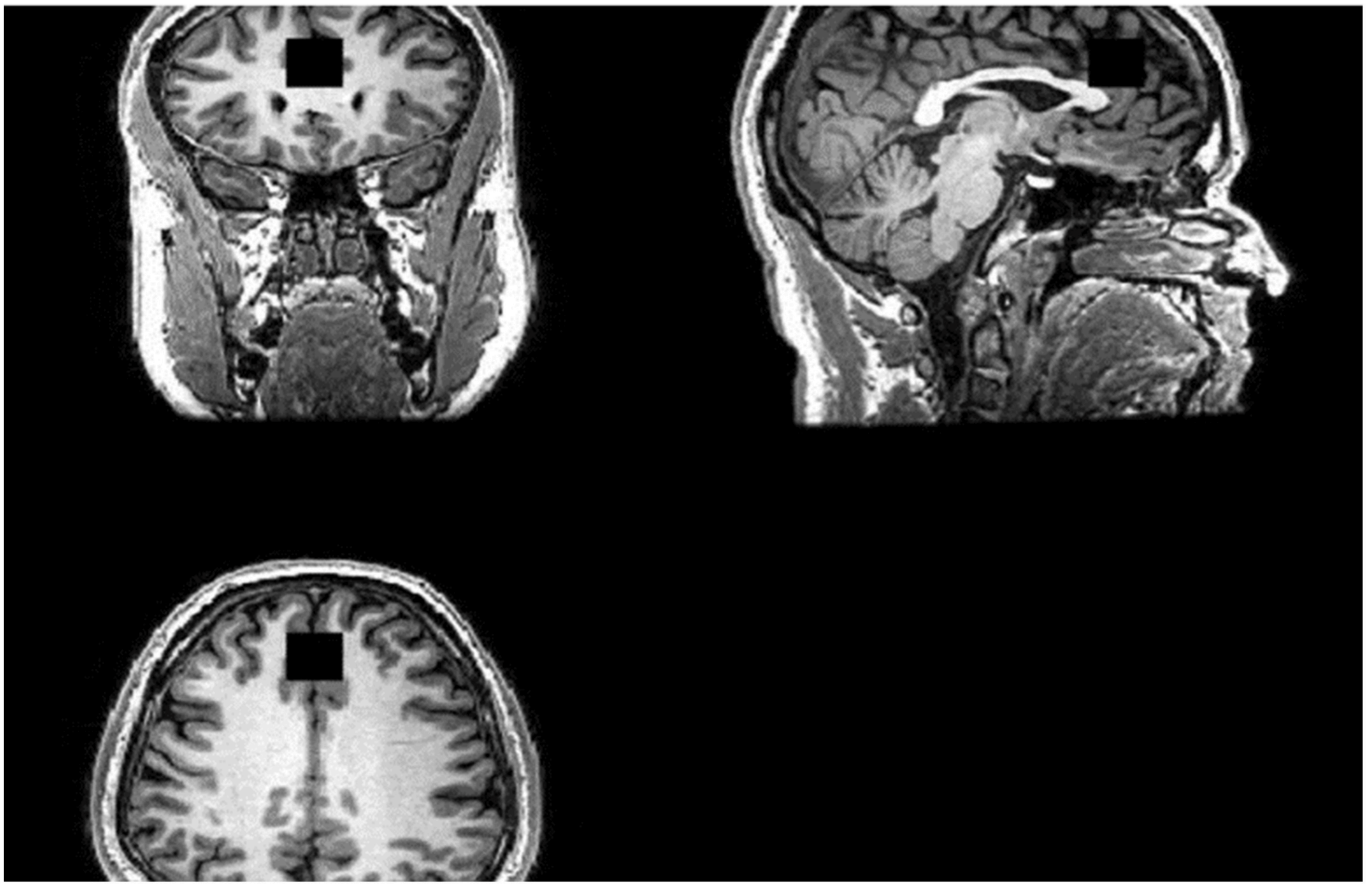
Region of Interest (ROI). ACC Voxel measures 20 (AP) × 20 (RL) × 20 (SI) mm3 in size and the voxel was prescribed from the midline sagittal localizer, with the center of the voxel placed 16 mm above the genu of corpus callosum perpendicular to the AC–PC line.

Scanning was performed on a GE 3-Tesla System (GE, 12-channel head coil). For image guidance and prescription of voxels of interest, axial T1 images were obtained using three-dimensional magnetization-prepared rapid gradient-echo with settings of time to repetition (TR) = 2,000 (ms), time to echo (TE) = 30 ms, time following inversion pulse (TI) = 1,100 ms, flip angle = 8° and voxel size 1 × 1 × 1 mm^3^. ^1^H-MRS was acquired using the standard PRESS sequence with the following parameters: TR = 2,000 ms, TE = 30 ms. See Figure 1 for our voxel of interest.

### ^1^H-MRS Analysis

^1^H-MRS analysis was accomplished using LCModel. The water-suppressed time-domain data were analyzed between 0.2 and 4.0 ppm. using the basis set provided by the vendor. T1-weighted images were segmented into gray matter, white matter and cerebrospinal fluid (CSF) compartments using Structural Brain Mapping (SPM) software on the whole brain T1 images. A volume of interest was generated on the T1 images, which matched the location and size of ^1^H-MRS voxel. Volumes of gray matter, white matter and CSF segments in this volume of interest were then calculated. Since the Glu metabolite measures were derived with water-scaling, a further correction was applied to correct the estimated water concentration of the voxel for partial volume CSF contamination. We used the same default CSF, gray matter and white matter water concentrations employed by LCModel (55,556, 43,300, and 35,880 mol/m^3^ respectively). For practical purposes, these correction factors were combined into a single equation M_corr_ = M^*^(43,300^*^gm + 35,880^*^wm + 55,556^*^CSF)/[35,880^*^(1 – CSF)], which simplifies to M^*^(1.207^*^gm + wm + 1.548 CSF)/(1 – CSF) where M = uncorrected metabolite, wm, white matter fraction; gm, gray matter fraction; and CSF, cerebrospinal fluid faction from the spectroscopy voxel.

### Immune Assessments

Blood was obtained in two EDTA tubes and were then immediately stored at −80 °C until batched assay. Concentrations of tumor necrosis factor (TNFa), interleukin 6 (IL_6), and C-reactive Protein (CRP) were assessed in duplicate using multiplex bead-based assays. Blood samples were originally collected from all individuals, however, at analysis stage, some samples could not be found on the sample management system, therefore we were left with 13 BP-II and 10 HC.

### Statistical Analysis

Pearson product-moment correlation coefficient was performed to assess the relationship between absolute Glu concentrations in the dorsal Anterior Cingulate Cortex (dACC) and cognitive tests (executive function, verbal learning and psychomotor speed). Secondary analyses examined the relationship between inflammatory markers and absolute Glu concentrations in the dACC. Independent samples T-tests were used to examine the differences in Glu, cognitive tests, and cytokines i.e., TNF-a, IL_6, and CRP levels between groups, as well as age, sex, hypomanic symptoms measured by YMRS and depression severity as measured by the MADRS. All results were assessed for multiple comparisons, using False Discovery Rate (FDR) analysis, in SPSS.

## Results

BP-II participants had significantly higher scores on YMRS and MADRS compared to controls (see Table 1). There was no significant difference between groups on WTAR. There was a trend for a significant difference between healthy controls and BP-II on the DST and no other significant differences between groups on other cognitive tests, however BP-II tended to perform less well. See Table 1. The BP-II group had significant psychiatric co- morbidity compared to healthy controls, see Table 2.

**Table 1:** Mean and standard deviations of clinical and cognitive characteristics.

	**Healthy control** ***n = 13***		**Bipolar disorder** ***n= 15***			
	***M***	***SD***	***M***	***SD***	***t-value***	***p-value***
YMRS	0.69	1.03	4.36	3.89	3.395	0.004
MADRS	0	0	9.43	12.42	−2.840	0.014
DST	90	18.3	73	24.5	2.005	0.052
RAVLT	56	9.7	51	39.6	1.706	0.996
TMT-B	60.78	36.9	60.85	28.1	−0.005	0.107
WTAR	45.67	4.812	46.21	3.017	−0.341	0.152

**Table 2:** Co-morbidities in the patient sample (BP-II).

**Diagnosis**	***N***	**%**
Suicide risk	8	50%
Panic disorder	6	43%
Agoraphobia	5	31%
OCD	1	6%
PTSD	3	12%
Alcohol dependence	1	6%
Alcohol abuse	1	6%
Psychosis	3	12%
Generalized Anxiety Disorder (GAD)	4	19%

### Bipolar Disorder Type II (BP-II) vs. Healthy Control

#### Glutamate (Corrected for CSF/Gray/White Matter)

There was no significant difference in anterior cingulate Glu levels between BP-II (*M* = 15.92, *SD* = 1.67) and HC (*M* = 15.17, *SD* = 1.50), *t*(−1.420, *p* = 0.227).

#### Inflammatory Markers

There were no significant differences in TNFa, IL_6, or CRP levels between BP-II and healthy controls. See Table 3.

**Table 3:** BP-II vs. healthy control—TNFa, IL_6, CRP.

	***N***	***Mean***	***SD***	***p-value***	***t-value***
**TNFa**					
Healthy control	10	1.7730	0.44242	0.221	−1.322
Bipolar disorder	13	2.0215	0.45284		
**IL_6**					
Healthy control	10	0.53	0.371	0.792	−0.267
Bipolar disorder	13	0.58	0.479		
**CRP**					
Healthy control	10	3.02	2.79	0.400	−0.871
Bipolar disorder	13	5.23	4.81		

#### TNFa, IL_6, CRP vs. Glu in BP-II

There were no significant relationships between TNFa (*r* = 0.103, *p* = 0.512), IL_6 (*r* = 0.211, *p* = 0.345), and CRP (*r* = 0.356, *p* = 0.435) with Glu levels in the BP-II group.

#### Glu vs. Cognitive Data in BP-II

There were no significant relationships between scores on the DST and Glu levels in patients (*r* = −0.436, *p* = 0.120) and no relationship between scores on TMT-B and Glu levels in the BP-II group (*r* = 0.194, *p* = 0.524). There were no significant correlations between Glu and RAVLT or WTAR.

#### Digit Symbol Task vs. Outcome

Scores on the DST were significantly associated with therapy treatment outcome following psychological therapy (*r* = −0.582, *p* = 0.037), i.e., worse performance on the DST is associated with higher depressive severity following treatment.

## Discussion

We found higher levels of CRP, IL_6 and TNFa and elevated levels of dACC Glu in bipolar patients in comparison to controls, however the differences were not significant. We found that BP-II subjects tended to perform worse in cognitive tests however the difference were not significant between groups. There was a trend for significance on the DST measure of processing speed (*p* = 0.052) between groups. In turn, we found that reduced processing speed was associated with a poor outcome to psychological treatment.

Our findings contribute to our understanding of neural correlates of Bipolar Disorders that are characterized as having impaired cognitive function (i.e., difficulties in attention and concentration are together, a hallmark of both major depression and hypomania).

## Limitations

The study was designed as a pilot, and as such has a small sample size. As a result, it is possible that some of the negative findings may have been due to a lack of power. Although all patients were assessed as euthymic at screening, three patients were depressed at the time of their scan. Strict euthymic samples should be investigated in further samples. The authors note that although no significant differences were found between groups, the lead author of this study extracted participants who were depressed on the day of the scan and the mean differences in Glu levels between patients and controls were larger, although again, not significantly so. The patients in this study were not excluded based on the use of anti-depressants, given the challenge of recruiting patients with BP-II without medication (12). Although 10/15 patients were not taking anti-depressants, the results could have been influenced by those who were on medication.

## Conclusions

Two evolving theories regarding the development of mood disorders involve excessive activation of inflammatory pathways and alterations in glutamate metabolism. In the present study, we did not find a significant comparison for the involvement of inflammation or in glutamate levels between groups. The reason for this may be in part due to the small sample size, especially as our findings show elevated levels of both cytokines and Glu, which fits with our hypothesis, although not significant. It could also mean that peripheral inflammation is not related to brain Glu in the dACC. Nevertheless, cognitive impairment, and particularly in the area of processing speed, appears to be an important and novel treatment target for improving neuropsychological function in BP-II groups. Improving such factors may lead to better outcomes to psychological therapy and related treatments.

Future work in our group will aim to further characterize the relationship between inflammation, mood and glutamatergic neurotransmission.

## Author Contributions

SK carried out data collection, all data analyses, and writing of the paper. LJ helped with data collection and preparation. CP provided support and expertise with inflammatory data. AC provided expert guidance and supervision on data analyses and write up. AY provided expert guidance and supervision on write up. JS provided expert guidance and supervision on data analyses and write up. CH helped with data collection and preparation.

### Conflict of Interest Statement

AY, AC, and JS: Employed by King's College London; Honorary Consultant SLaM (NHS UK); Paid lectures and advisory boards for all major pharmaceutical companies with drugs used in affective and related disorders; No share holdings in pharmaceutical companies; Investigator initiated studies from AZ, Eli Lilly, Lundbeck, Wyeth; Grant funding (past and present): NIHR-BRC (UK); NIMH (USA); CIHR (Canada); NARSAD (USA); Stanley Medical Research Institute (USA); MRC (UK); Wellcome Trust (UK); Royal College of Physicians (Edin); BMA (UK); UBC-VGH Foundation (Canada); WEDC (Canada); CCS Depression Research Fund (Canada); MSFHR (Canada); NIHR (UK). CP is funded by the UK National Institute for Health Research (NIHR) Biomedical Research Center at the South London and Maudsley NHS Foundation Trust and King's College London, the UK Medical Research Council (grants MR/L014815/1, MR/J002739/1 and MR/N029488/1) and the Psychiatry Research Trust. The remaining authors declare that the research was conducted in the absence of any commercial or financial relationships that could be construed as a potential conflict of interest.
